# Neonatal body composition, salivary feeding gene expression, and feeding outcomes in infants of diabetic mothers

**DOI:** 10.3389/fcdhc.2024.1501805

**Published:** 2024-12-19

**Authors:** Dara Azuma, Yvette Penner, Tomoko Kaneko-Tarui, Taysir Mahmoud, Janis L. Breeze, Angie Rodday, Perrie O’Tierney-Ginn, Jill L. Maron

**Affiliations:** ^1^ Mother Infant Research Institute at Tufts Medical Center, Boston, MA, United States; ^2^ Frances Stern Nutrition Center at Tufts Medical Center, Boston, MA, United States; ^3^ Clinical and Translational Science Program, Tufts University Graduate School of Biomedical Sciences, Boston, MA, United States

**Keywords:** maternal diabetes, neonatal, saliva, feeding, body composition

## Abstract

**Introduction:**

Infants of diabetic mothers (IDMs) may exhibit decreased oral intake, requiring nasogastric feedings and prolonged hospitalization. The objective of this study was to explore whether saliva serves as an informative biofluid for detecting expression of hunger signaling and energy homeostasis modulator genes and to perform exploratory analyses examining expression profiles, body composition, and feeding outcomes in late preterm and term IDMs and infants born to mothers with normoglycemia during pregnancy.

**Methods:**

In this prospective cohort pilot study, infants born at ≥ 35 weeks’ gestation to mothers with gestational or type II diabetes (IDM cohort) and normoglycemic mothers (control cohort) were recruited. The presence of known hunger signaling genes: 5’AMP-activated protein kinase (*PRKAA2*) and neuropeptide Y2 receptor (*NPY2R*); adipokines: leptin *(LEP*) and adiponectin *(ADIPOQ)*; and energy homeostasis regulators: ghrelin *(GHRL)* and proopiomelanocortin *(POMC)* in neonatal saliva was determined with RT-qPCR and compared between cohorts. Body composition was assessed via skinfold measurements and compared between cohorts. Feeding outcomes were recorded. Exploratory analyses were performed examining associations between infant body composition, energy homeostasis and hunger signaling gene expression.

**Results:**

Twenty-three infants in the IDM cohort and 22 infants in the control cohort were recruited. *LEP* and *ADIPOQ* were not reliably detected in neonatal saliva in either cohort. *PRKAA2, GHRL* and *NPY2R* were less likely to be detected in the IDM cohort, whereas *POMC* was more likely to be detected in the IDM cohort. Infants in the IDM cohort had greater adiposity compared to infants in the normoglycemia cohort. Only 3 IDMs had documented poor feeding; no infant in the control group struggled to feed. In exploring associations between hunger signaling gene expression with energy homeostasis gene expression and body composition, the odds of detecting salivary *NPY2R* expression decreased as fat mass increased, and the odds of detecting *PRKAA2* expression increased in the presence of *GHRL* expression.

**Discussion:**

Non-invasive assessment of hunger signaling and energy homeostasis gene expression is possible through neonatal salivary analysis. This pilot study lays the foundation for a larger scale study to further investigate the link between *in utero* exposure to diabetes with body composition and regulation of appetite.

## 1 Introduction

**Figure 1:**
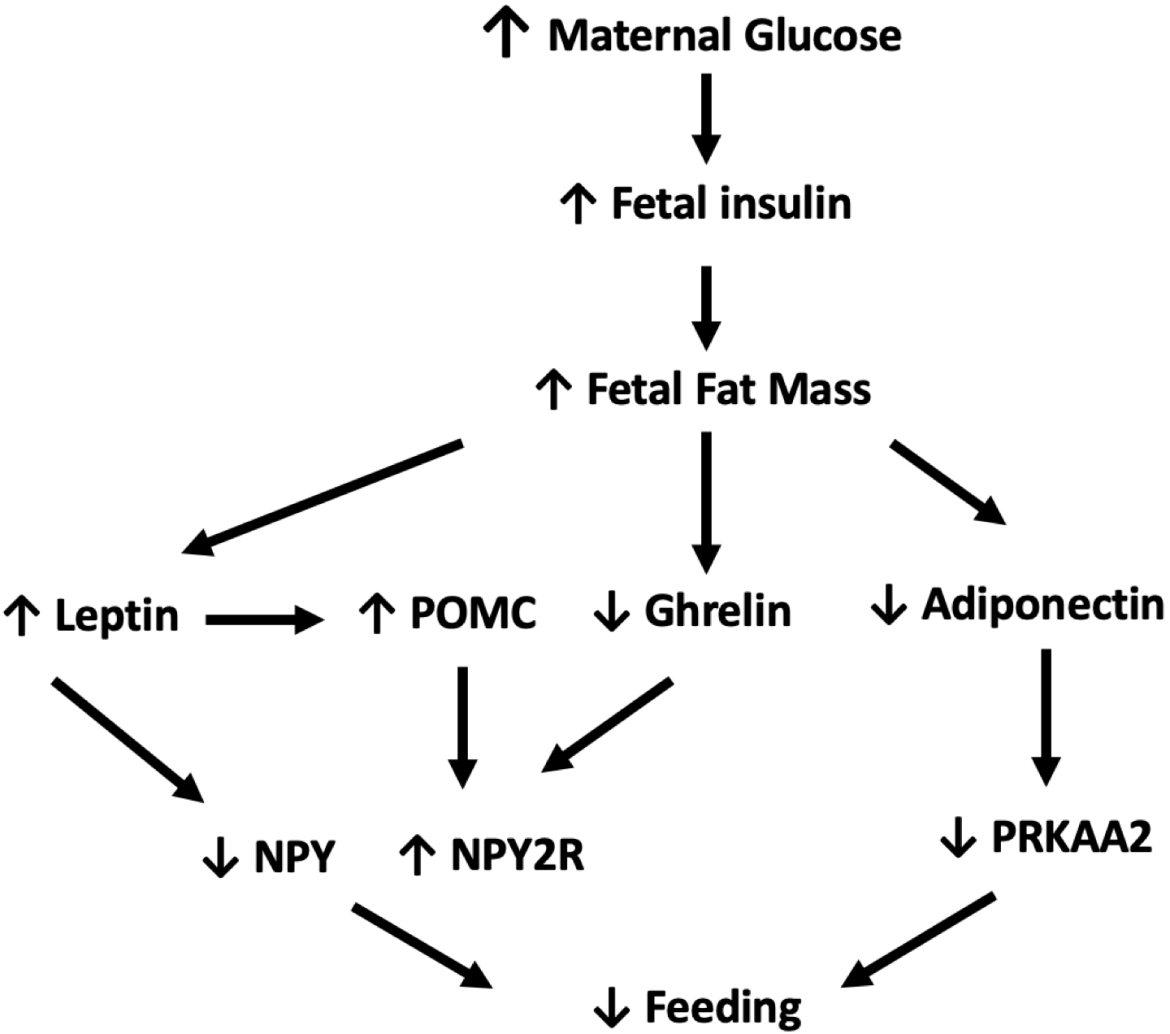
In response to elevated maternal glucose levels, fetal insulin levels and subsequent fetal fat mass increase. In turn, elevated levels of leptin simultaneously increase *POMC* while decreasing *NPY* expression. Increased fetal fat mass also downregulates ghrelin and adiponectin with resulting decrease in expression of *PRKAA2* and upregulation of *NPY2R*. Combined these pathways work in synergy to decrease oral intake. ↓:decrease, ↑:increase, POMC, proopiomelanocortin; PRKAA2, 5'AMP-activated protein kinase; NPY, Neuropeptide Y; NPY2R, Neuropeptide Y 2 receptor (9).

In 2023, the Centers for Disease Control and Prevention reported that the percentage of pregnant women diagnosed with gestational diabetes (GDM) rose from 6.0% in 2016 to 8.3% in 2021 (1–3). Additionally, there was a 37% increase in the number of women diagnosed with type I and type II diabetes mellitus (DM) prior to pregnancy between 2000-2010 (3). As the prevalence of pre-gestational diabetes and GDM rises, research has focused on the short- and long-term implications of *in utero* exposure to diabetes on infants (4–7). Poor oral feeding in infants of diabetic mothers (IDMs) is an important, but not well studied, short-term complication of exposure to high levels of glucose *in utero* (5). IDMs who exhibit decreased oral intake may require nasogastric feedings and prolonged hospitalization (8). There is a limited body of literature describing the epidemiology and pathophysiology of poor oral feeding in IDMs, and only a few studies have examined the root cause of feeding issues in this population. One study found that IDMs have immature sucking patterns secondary to neuroimmaturity (5) and another determined that IDMs have a maladaptive vagal neuropathy resulting in increased gastroesophageal reflux (7). Both mechanisms are suspected to lead to poor feeding in this population (5, 7). We proposed an alternative explanation that poor oral intake in the neonatal period may be the result of decreased appetite (**Figure 1**).

It is well known that IDMs have greater gestational age-adjusted birthweights and adiposity as compared to infants born to mothers with normal glucose tolerance during pregnancy (10–12). Adipose tissue is an active endocrine organ that modulates energy expenditure, appetite, and glucose homeostasis through the help of adipokines (adiponectin and leptin), as well as ghrelin and proopiomelanocortin (POMC) (13–18). Adiponectin is a fat-derived hormone whose levels are inversely proportional to the amount of fat tissue present (15, 19). Leptin acts as a satiety signal and counter regulatory hormone sent from peripheral fat mass to the hypothalamus (20). Leptin is thought to play a role in the pathologic growth of infants of gestational diabetic mothers and is considered a marker of fetal adiposity (19). Leptin increases pro-opiomelanocortin (POMC) gene expression (14, 21). POMC neurons play a role in both glucose homeostasis and energy balance. POMC is elevated in obesity and decreased in fasting states (22). Ghrelin has been shown to stimulate food intake in animal models and humans (23, 24). Several studies have shown changes in the adipoinsular axis, as a result of *in utero* exposure to hyperglycemia, which leads to fetal overgrowth (16, 19, 20). Adipokines, ghrelin, and POMC alter appetite through neuropeptide Y(NPY) and 5’AMP-activated protein kinase (PRKAA2) (14, 15, 23). Salivary gene expression of *PRKAA2* and neuropeptide Y2 receptor (*NPY2R)* has been shown to be detected in preterm and term neonates. *PRKAA2* demonstrates decreased or undetectable levels in unsuccessful oral feeders; presence or increased expression of *NPY2R* has been reported in unsuccessful oral feeders (25, 26).

To date, research in this area has largely focused on cord blood and serum levels of leptin, ghrelin, and adiponectin in large for gestational age infants, IDMs, and their normoglycemic counterparts (16, 19, 20). One study compared cord plasma and salivary levels of insulin and leptin in infants and found a positive correlation, although not statistically significant (27). However, to the best of our knowledge, no study has examined salivary gene expression profiles of these biomarkers in neonates. Furthermore, these levels have not been measured with respect to feeding or hunger signaling gene expression in IDMs.

The objective of this study was to explore whether saliva serves as an informative biofluid for detecting expression of hunger signaling and energy homeostasis modulator genes and to perform exploratory analyses correlating expression profiles with feeding outcomes and body composition in late preterm and term IDMs and infants born to mothers with normoglycemia during pregnancy.

## 2 Methods

### 2.1 Study design and study population

In this prospective cohort pilot study, subjects were recruited from Tufts Medical Center in Boston, Massachusetts between May 2019 and September 2021. Eligible infants were born at ≥ 35 weeks’ gestation. Infants with a birth weight < 10^th^ percentile (28), or with a history of intrauterine growth restriction, maternal diagnosis of preeclampsia or type I DM, or major congenital or chromosomal anomalies were excluded to avoid infants at risk for fetal growth restriction or placental insufficiency that may have skewed the data. Infants with fetal growth restriction may have a different physiologic response due to the paucity of nutrition *in utero*. Infants with *in utero* opioid exposure were also excluded based on prior research in this population showing alterations in hunger signaling and hypothalamic gene expression (29). We studied two cohorts of infants—IDMs and infants born to mothers with normal glycemic tolerance during pregnancy. Infants in the diabetic cohort were defined as infants born to mothers with GDM during the current pregnancy or a history of type II DM. Infants in the normoglycemic cohort were born to mothers with normal glucose tolerance testing during pregnancy.

Study eligibility was assessed upon maternal admission to the labor and delivery unit or while the infant was admitted to the newborn nursery or neonatal intensive care unit. All study data, measurements and samples were collected during the infant’s hospitalization. Maternal pregnancy information and demographic characteristics, as well as infant growth parameters, glucose levels when acquired, medications, and feeding data were extracted from the medical record. Infants were characterized as having feeding issues if there was documentation in the medical record about feeding concerns, such as prolonged hospitalization for suboptimal oral intake in the newborn nursery or requiring nasogastric tube for feeding due to poor oral intake. Since infant sex may influence salivary gene expression of hypothalamic and hunger signaling genes (29), efforts were made to recruit an equal number of male and female infants in each cohort. This study was reviewed and approved by the IRB at Tufts Medical Center. Written informed consent was obtained from a parent or guardian prior to infant participation in this study.

### 2.2 Infant anthropometric and body composition measurements

Infant length was measured to the nearest 0.1 cm using an infant length board. Two measurements were taken and if the difference between the two measurements was greater than or equal to 0.5 cm, a third measurement was obtained. The infant’s length was recorded as the average of all measurements. Skin fold thickness at the left hip was measured using calibrated skinfold calipers (Harpenden) and was measured to the nearest 0.1 mm. Two measurements were taken and if the difference between the two measurements was greater than or equal to 0.5 mm, a third measurement was obtained. Infant weight, length, and skinfold measurements were inputted into the Catalano formula to calculate fat mass in kg and percent fat mass (30). Percentiles were calculated for infant fat mass and percent fat mass taking into account infant sex and gestational age at birth using body composition curves published by Norris, et al., 2019 (31). Infant birth weight percentiles also took into account infant sex and gestational age using the Olsen, 2010 growth curves (28, 32). Infant weight was measured using a bedside scale and extracted from the medical record.

### 2.3 Salivary gene expression

Gentle suctioning of the oropharynx was performed to collect two infant salivary samples anytime during the birth hospitalization. To minimize breast milk and associated maternal RNA contamination, saliva was collected prior to feeding when possible. The samples were placed in an RNA stabilization solution and were stored until salivary RNA extraction was performed using RNeasy Micro Kit (Qiagen, Hilden, Germany) per manufacturer’s instructions. On column DNase treatment was performed using RNase-free DNase I Set (Qiagen, Hilden, Germany) to minimize genomic contamination. Samples were then converted to complementary DNA (cDNA) using SuperScript VILO cDNA Synthesis Kit (Invitrogen, Carlsbad, California) and underwent targeted preamplification using TaqMan PreAmp Master Mix Kit (Applied Biosystems, Foster City, California). Detailed methods for salivary sample processing are outlined in the methods section of Yen, et al., 2019 (29).

Quantitative real-time polymerase chain reaction (qPCR) on cDNA was performed to relatively quantify gene expression of *NPY2R, PRKAA2*, leptin (*LEP)*, ghrelin *(GHRL), POMC* and adiponectin (*ADIPOQ)* as previously described by Yen, et al., 2019 (29). Samples were run on Custom TaqMan Array qPCR plates (Applied Biosystems, Foster City, California). Each plate was run with a control human mRNA sample provided by Applied Biosystems. Reference genes (*YWHAZ*, *GAPDH*, and *HPRT1*) demonstrated by Khanna et al. to have stable expression across sex and gestational age, were quantified for each sample (33). Genes of interest (*NPY2R, PRKAA2, LEP, GHRL, POMC* and *ADIPOQ)* were run in duplicate for each patient sample. Amplification of all 3 reference genes as an assessment of RNA integrity in each sample was a prerequisite for inclusion of samples in the final analysis. If any reference gene failed to amplify, the secondary sample was analyzed as described above. A negative water control was run once to ensure there were no unintended amplification products (primer dimers).

### 2.4 Statistical analysis

Data analysis was conducted using R version 4.0.2 (R Foundation for Statistical Computing, Vienna, Austria). Maternal and infant demographics were summarized using means and standard deviations for parametric continuous data and medians and interquartile ranges for non-parametric continuous data. Salivary gene expression was treated as a binary variable. If cycle threshold (Ct) for amplification was met by 40 cycles, the gene was noted to be present in the saliva sample. If the Ct was not met by 40 cycles, the gene did not amplify and was deemed to be absent in the saliva sample. Salivary gene expression and body composition were summarized by cohort and feeding outcome. Salivary gene expression was compared between our 2 study cohorts using Fisher’s exact test. Body composition measurements were compared between infants born to diabetic and normoglycemic mothers using the Wilcoxon rank sum test. Univariate logistic regression analyses using binary hunger signaling gene expression of *PRKAA2* and *NPY2R* as the outcome variable were run to explore associations between infant body composition or expression of energy homeostasis regulators (*POMC* and *GHRL*) and hunger signaling genes independent of study cohort. Given the nature of a pilot study with inherent limitations of sample size, regression analyses were done to provide estimates and variability.

## 3 Results

### 3.1 Study population characteristics

A total of 45 infants were recruited, with 22 infants in the normoglycemia group and 23 in the diabetic group. Mothers diagnosed with diabetes were older and had higher pre-pregnancy BMIs as compared to mothers with normoglycemia during pregnancy. IDMs had lower gestational ages at birth and higher birth weight for age percentiles (28) as compared to the normoglycemia cohort. There were no infants in the normoglycemia cohort with feeding issues and only 3 infants (13%) in the diabetic cohort with feeding issues noted in the medical record (**Table 1**).

**Table 1:** Maternal and infant characteristics according to maternal diabetes status.

	Normoglycemia Cohort (n=22)	Diabetic Cohort (n=23)	p-value
Maternal Age at delivery (years)	30.4 (4.9)	34.3 (3.5)	<0.01
Maternal Pre-pregnancy BMI (kg/m^2^)	24.5 (21.2, 32.9)	31.7 (28.5, 33.9)	0.01
Male	14 (63.6)	11 (47.8)	0.44
Gestational age at delivery (weeks)	39.4 (39.1, 39.8)	37.9 (37.2, 39.0)	<0.01
Birth weight (kg)	3.4 (0.3)	3.4 (0.5)	0.86
Birth percentile by gestational age*	50.0 (30.5, 61.3)	59.0 (43.5, 88.5)	0.05
Feeding Issues	0 (0)	3 (13.0) ^	0.25

*Birth Percentile calculated using Olsen 2010 growth curves (28).

Data are presented as mean (standard deviation) or median (interquartile range) for continuous measures, and n (%) for categorical measures.

### 3.2 Salivary gene expression

Salivary samples were collected once from all 45 infants and collection time ranged from day of life 0 to 7. Three samples from the diabetic cohort did not amplify one of the three reference genes, and so the secondary sample was processed, which passed quality control. *LEP* was only able to be quantified in three samples in the normoglycemia cohort and one sample in the diabetic cohort. *ADIPOQ* was only detectable in one sample in the normoglycemia cohort. *PRKAA2, GHRL*, and *NPY2R* were less likely to be detected in the diabetic cohort, whereas *POMC* was more likely to be detected in the diabetic cohort (**Table 2**). There was no gene expression detected for the negative water control. The positive control had minimal plate-to-plate variability with respect to the geomean of the three reference genes (mean 23.7± 0.4).

**Table 2:** Presence of individual gene expression in saliva and body composition according to maternal diabetes status.

	Normoglycemic Cohort (n=22)	Diabetic Cohort (n=23)	p-value
*ADIPOQ*	1 (4.5%)	0 (0.0%)	0.49
*PRKAA2*	20 (90.9%)	19 (82.6%)	0.67
*GHRL*	18 (81.8%)	18 (78.2%)	1.00
*LEP*	3 (13.6%)	1 (4.3%)	0.35
*NPY2R*	19 (86.3%)	19 (82.6%)	1.00
*POMC*	16 (72.7%)	17 (73.9%)	1.00
Fat Mass (g)	364.3(132.0)	372.2(181.7)	0.87
Percent Fat Mass	11.0 (3.2)	11.3 (3.9)	0.96
Percent Fat Mass Percentile	59.7 (26.8)	67.1 (28.1)	0.52

Data are presented as mean (standard deviation), and n (%) for categorical measures.

### 3.3 Body composition measurements

Body composition measurements were collected once from all 45 infants and collection time ranged from day of life 0 to 7. Infants born to mothers with diabetes had a slightly higher absolute fat mass as well as percentage of fat mass as compared to infants born to mothers who were normoglycemic, despite having a lower gestational age at birth. When looking at body composition percentiles by gestational age and sex using body composition growth curves (31), infants in the diabetic cohort had a greater fat mass percentile compared to infants born to mothers who were normoglycemic during their pregnancy (**Table 2**).

### 3.4 Feeding outcomes and exploratory analyses

Three infants in the diabetic cohort had poor feeding documented in their medical record, while none in the control group had documented feeding issues. These infants were eventually able to eat by mouth and were discharged home. No additional work up was required for poor feeding. Generally, infants with documented feeding issues were born at a lower gestational age, with a median age of 37.6 weeks as compared to infants without feeding issues, with a median age of 39.1 weeks. Poor feeders had a greater birth weight of 3.57 kg and birth percentile of 72, as compared to feeders who had a median birth weight of 3.38 kg and birth percentile of 50.5. The poor feeders also had a greater fat mass of 0.45 kg and percentage of fat of 14.2, as compared to infants without feeding issues which was 0.33 kg and 10.3 respectively. Feeders were more likely to express *GHRL*, *NPY2R* and *POMC* and less likely to express *PRKAA2* than non-feeders. Greater infant adiposity was associated with lower odds of expression of *NPY2R.* Expression of *GHRL* was associated with increased odds of expression of *PRKAA2* (**Table 3**).

**Table 3:** Univariate logistic regression for associations between hunger signaling gene expression with body composition and energy homeostasis gene expression.

Univariate	*PRKAA2*		*NPY2R*	
	Odds Ratio	95% CI	Odds Ratio	95% CI
Fat Mass (per 100 g)	0.18	0.36, 1.09	0.53	0.29, 0.89
Percent Fat Mass	0.83	0.64, 1.06	0.76	0.57, 0.97
*GHRL*	13.60	2.11, 119.71	4.00	0.65, 23.30
*POMC*	3.33	0.54, 20.99	1.12	0.14, 6.18

## 4 Discussion

### 4.1 Salivary gene expression

This pilot study was able to demonstrate the feasibility of detecting *PRKAA2, NPY2R, GHRL*, and *POMC* in saliva of our study population, but we were unable to reliably detect *LEP* and *ADIPOQ*. These results do not necessarily mean that leptin and adiponectin are not expressed in infant saliva, as there may be more sensitive assays that could detect their gene expression. It is important to consider whether one should target gene expression for the hormone of interest or the receptor. While we were unable to detect *LEP* in our study, prior studies have detected *LEPR* expression in neonatal saliva (29). Receptor expression may be better quantified in saliva. We did not measure serum levels of target hormones for comparative analysis.

Neonatal saliva is a non-invasive biofluid that contains a wealth of information which can be collected serially with minimal impact on the infant (25). Salivary diagnostics may be helpful in not only identifying infants at risk for poor feeding, but more importantly, elucidating disruption in regulatory pathways responsible for outcomes. The salivary transcriptome also allows for non-invasive serial monitoring of changes in energy regulators and hunger signaling over time.

*PRKAA2, GHRL*, and *NPY2R* were less likely to be detected in the diabetic cohort, whereas *POMC* was more likely to be detected in the diabetic cohort. Decreased expression of *PRKAA2* and *GHRL* and increased expression of *POMC* is consistent with biological mechanisms relating these hormones to adiposity, decreased oral intake and poor feeding outcomes. *Post-hoc* power analysis yielded a power of 3 to 12 percent, given the small sample size.

### 4.2 Body composition measurements

Infants in the diabetic cohort had greater overall fat mass and percent fat mass despite being born at a younger gestational age. It has been shown that body fat mass and body fat percentage increases as gestational age increases and females have greater adiposity than males (31). To account for changes in body composition with gestational age and sex, Norris et al (31) compiled body composition data from four studies in the United States and Ireland to create body composition curves stratified by percentile. Our cohort generally had lower fat mass, but a greater percentage of fat mass to total body weight, as compared to the cohort used to derive the body composition curves. This may be due to differences in modalities used to measure body composition, as we used anthropometric measurements and skin fold thickness rather than air displacement plethysmography. Future studies may consider adjusting for infant sex and gestational age when interpreting body composition measurements or using body composition curves, although care should be taken in considering how body composition data are obtained.

### 4.3 Study strengths and limitations

Our study utilized non-invasive methods to investigate the relationship between body composition and regulation of appetite through adipoinsular and hypothalamic hormones in infants of diabetic mothers. By demonstrating the feasibility of salivary gene expression analysis of these pathways, future investigators can study how these pathways change over time. This method may be preferred over cord blood which is also non-invasive but only provides information on the *in utero* environment, as well as serial blood draws which are challenging to obtain and inflict harm. Since neonatal normative serum gene expression levels for our target hormones do not exist, we need not perform a comparative analysis between serum and saliva to determine a gold standard biofluid for measurements. Rather, we aimed to determine if levels in saliva would be independently informative of feeding outcomes. While it is possible that salivary gene expression is impacted by the oral microbiome and metabolome, it was beyond the scope of this study to determine what if any impact these would have on gene expression (34).

One limitation of our study was that maternal glucose control and treatment with medications during pregnancy in the diabetic mothers was not quantified but may be an important factor related to infant adiposity, energy homeostasis, and hunger signaling. It may also be informative to separate infants born to mothers with pregestational versus gestational diabetes, as the duration of exposure to an elevated glucose concentration varies between the groups. However, we were unable to do so given our sample size. Moreover, placental nutrient metabolism and delivery impact (35) on the newborn is outside the scope of the current project. An additional limitation of this study is that only three infants were noted to have feeding difficulties, which is far fewer than the 37% of IDMs cited in prior literature (36). It will be important for future studies to specifically target IDMs who struggle to feed, as these infants may be more likely to have dysregulation of their hypothalamic signaling and/or energy expenditure pathways directly impacting feeding success.

## 5 Conclusions

In conclusion, our study proposes a new explanation for poor feeding in IDMs through adipo-insular and hypothalamic regulation of appetite. Given ongoing increases in pre-gestational and GDM in the U.S., this is an important topic of study with serious short and long-term implications for the offspring of mothers with diabetes during pregnancy. We were able to demonstrate that it is feasible to detect both hunger signaling and energy metabolism genes in infant saliva to study the association between maternal diabetes, infant adiposity, and gene expression. Infant saliva provides a non-invasive means to serially monitor hormonal regulation of appetite and energy which can be used in future studies. Our results are descriptive but will hopefully encourage the design and conduct of a larger scale study that will systematically examine the relationship between body composition and adipoinsular regulation of hunger in infants of diabetic mothers who struggle to feed. By further elucidating changes in energy and appetite regulation in IDMs, we can better understand the mechanisms behind feeding issues to guide management of these infants.

## Data Availability

The data presented in the study are deposited in the OSF repository, accession number DOI 10.17605/OSF.IO/R3FKH, https://osf.io/r3fkh/.
